# The prevalence and clinical relevance of cervical abnormalities after an amputation of the cervix as part of prolapse surgery: a cross-sectional study

**DOI:** 10.1007/s00404-025-08135-y

**Published:** 2025-07-24

**Authors:** Véronique V. van Cooten, Dennis van Hamont, Albert G. Siebers, Luthy Alcalá, Ruud L. M. Bekkers, Leonie Speksnijder

**Affiliations:** 1 Department of Obstetrics and Gynaecology, Division of Urogynecology, Amphia Hospital, Breda, The Netherlands; 2 Palga, The Dutch Nationwide Pathology Databank, Houten, The Netherlands; 3 Department of Pathology, Amphia Hospital, Breda, The Netherlands; 4 Department of Obstetrics and Gynaecology, Catharina Hospital, Eindhoven, The Netherlands; 5 Department of Gynecologic Oncology, Radboudumc Nijmegen, Nijmegen, The Netherlands; 6 GROW School for Oncology and Reproduction, Maastricht University, Maastricht, The Netherlands

**Keywords:** Uterine Cervical Dysplasia, Uterine Cervical Neoplasm, Histology, Unnecessary Procedures, Pelvic Organ Prolapse, Gynecologic Surgical Procedures

## Abstract

**Purpose:**

To examine the safety of omitting routine histopathological examination by determining the prevalence of cervical pathology in women after cervical amputation as part of pelvic organ prolapse (POP) surgery without pre-existing indication for histology and the necessity of additional treatment.

**Methods:**

A cross-sectional study was performed using data of women who underwent cervical amputation as part of POP without pre-existing indication for histopathological examination, obtained from Palga, the Dutch nationwide pathology databank, between January 1991 and January 2022.

**Main outcome measures:**

The prevalences of the following histological diagnoses were determined: Cervical Intraepithelial Neoplasia (CIN I–III), adenocarcinomas in situ (AIS), cervical carcinomas, and other malignancies.

**Results:**

In total, 14.887 patients were included in this study, with a median age of 61.4 years (SD = 11.7). The prevalence of CIN II+ lesions was 6.9 [95%-CI 5.6, 8.3] per 1000 women, while one cervical carcinoma (6.7 [95%-CI −0.6, 19.9] per 100.000 women) was reported (stage IA1 microinvasive squamous cell carcinoma).

**Conclusion:**

This study found a prevalence of 0.7% for CIN II+ incidental findings in women undergoing amputation of the cervix as part of POP surgery. No additional treatments were required after the final histopathological results.

The decision to omit routine histopathological examination could potentially be safe, offering the prospect of reduced healthcare costs and environmental impact. Healthcare professionals should individually assess the risks and benefits of omitting and/or replacing routine histopathological examination.

## What does this study add to the clinical work

**Table:** 

Incidental CIN II+ findings were found in 0.7% after prolapse surgery. No additional treatments were required. Omitting routine histopathology could potentially be safe.	

## Introduction

Pelvic organ prolapse (POP) is a common condition in women [1], and its prevalence is expected to increase even further [2, 3]. POP can be managed both conservatively and surgically [3]. Although the exact prevalence of POP is unknown presumably due to underreporting, the lifetime risk of POP surgery has been reported in up to twenty percent of all women [2–5]. A wide variety of surgical techniques are available, ranging from hysterectomies to uterus-sparing techniques, e.g. modified Manchester operation or sacrospinous hysteropexy [6].

After POP surgery, cervices are often routinely sent for histopathological examination. This routine histopathological examination could detect occult cervical intraepithelial neoplasia (CIN) grade I-III, adenocarcinomas in situ (AIS), and cervical malignancies. Depending on the histopathological results, further treatment and/or staging may be indicated [7, 8].

The management of premalignant cervical abnormalities ranges from watchful waiting to excision or conisation [8]. For early stage cervical cancer, conisation or cervical amputation may suffice; however, more advanced stages may require more extensive surgery or even radiation therapy with or without chemotherapy [9]. Further treatment after a modified Manchester-Fothergill procedure, which includes amputation of the cervix, would only be necessary in case of cervical carcinomas stage IA2 and more [1, 10].

In the Netherlands, a cervical cancer screening program is conducted among women between 30 and 60 years old [11]. Monitoring of this nationwide program showed both a decrease in CIN detection rates as well as in the proportion of CIN II+ lesions above 60 years [12]. POP surgery is more frequently performed among these older groups, with the majority being over 60 years old [13]. The lower prevalence of CIN II+ lesions in this population, together with a reported low risk of cervical malignancy development after cervical POP surgery, questions the necessity of routine histological examination of cervices as part of POP surgery [14].

With increasing health care costs, lack of personnel and financial resources, together with the high frequency of POP surgery, further investigation on the need of routine histological examination is justified. Similarly, routine histopathological examination has been questioned for gallbladders after cholecystectomy, for nasal polyps in endoscopic sinus surgery, and for hemorrhoids and appendices after appendectomy [15–17].

The aim of our study was to examine the safety of omitting routine histopathological examination by determining the prevalence of cervical pathology in women after cervical amputation as part of POP surgery without pre-existing indication for histopathological examination and additionally determining the necessity of additional treatment.

## Methods

### Study population and design

A cross-sectional study was performed using data obtained from Palga, the Dutch Nationwide Pathology Databank [18]. The inquiry contained patients from the Palga database who underwent amputation of the cervix (e.g. Manchester Fothergill), resection or excision combined with prolapse surgery indicated in the clinical data on the application form.

We reviewed the provided clinical information on the application form and the conclusion of the histological reports. Patients with multiple histopathological specimens in the database, e.g. revised histopathology, were included once.

The remaining cases were categorized based on the type of procedure. Procedures other than the amputation of the cervix were excluded, e.g. hysterectomies, loop excisions, cervical conisations, cervical biopsies, unknown types of procedures, procedures with indications other than POP.

Furthermore, the conclusions of cyto- and histological reports before surgery were assessed. Patients with pre-existing indications for pathological examination were excluded, see Table 1. Exclusion was based on abnormal histo- and cytopathology reports before surgery without normalization, abnormalities on ultrasound, clinical suspicion of malignancy, abnormal vaginal blood loss, etc., see Table 1.

**Table 1:** Criteria pre-existing indications for histology

• Previously diagnoses high-grade cervical smear results within the past two years
• CIN I, II or III diagnosed in the past two years, without normalisation
• Cervical malignancy in patient history
• Clinal suspicion of malignancy
• History of vulvar carcinoma without (known) normal cervical smears
• Abnormal endometrial histology before surgery, e.g. hyperplasia with atypia
• Endometrial thickening >4 mm measured on ultrasound
• Abnormal vaginal blood loss, e.g. postmenopausal blood loss or postcoital bleeding
• Hereditary gynaecological carcinomas, genetic disorder or family history of gynaecological carcinomas
• Macroscopic or ultrasonic abnormality

### Analysis

The data of the remaining women who underwent cervical POP surgery without pre-existing indication for histopathological examination of the removed specimen were analyzed.

The detection rates of the following (pre-)malignant histological diagnoses were determined: CIN I-III, cervical carcinomas, and other gynecological malignancies. Data regarding CIN III lesions presented in this article also included AIS of the endocervix. Moreover, the detection rate of CIN II+ lesions, defined as CIN II, CIN III, and cervical malignancies, was calculated. We calculated the prevalence and 95% confidence intervals (95%-CI) using One-Sample T-Tests. Numbers needed to treat (NNT) were obtained by dividing 1 by the prevalence. Furthermore, we used FIGO staging of the cervix uteri (2018) to stage cervical carcinomas [7].

Age is presented as mean with standard deviation (SD). All statistical analyses were performed using SPSS version 28.0.1.0 for Windows (SPSS, Chicago IL).

### Ethical approval

This study was exempt from medical ethical approval because there was no physical involvement of the women in the study, and data were not traceable to individual participants. The study was approved by the scientific committee of Palga (lzv2020-3).

## Results

A total of 26.055 cases between January 1991 and January 2022 were initially selected. These cases underwent eligibility assessment. Five of these cases involved non-gynecological patients. Additionally, *n* = 28 cases were excluded as duplicates for the same patient. The remaining patients (*n* = 26.022) were categorized by type of surgical procedure. Subsequently, *n* = 10.998 patients were excluded due to procedure types outside the scope of this study. Additionally, *n* = 137 patients were excluded for having pre-existing indications for histopathological examination: *n* = 32 for abnormal cervical smears, *n* = 35 for CINs, *n* = 12 for carcinomas, *n* = 9 for endometrial hyperplasia, *n* = 43 for abnormal vaginal blood loss (incl. postmenopausal bleeding), *n* = 3 for macroscopic abnormalities, *n* = 2 for hereditary malignancies and *n* = 1 patient with a history of vulvar carcinoma without known cervical smears follow-up.

**Fig. 1:**
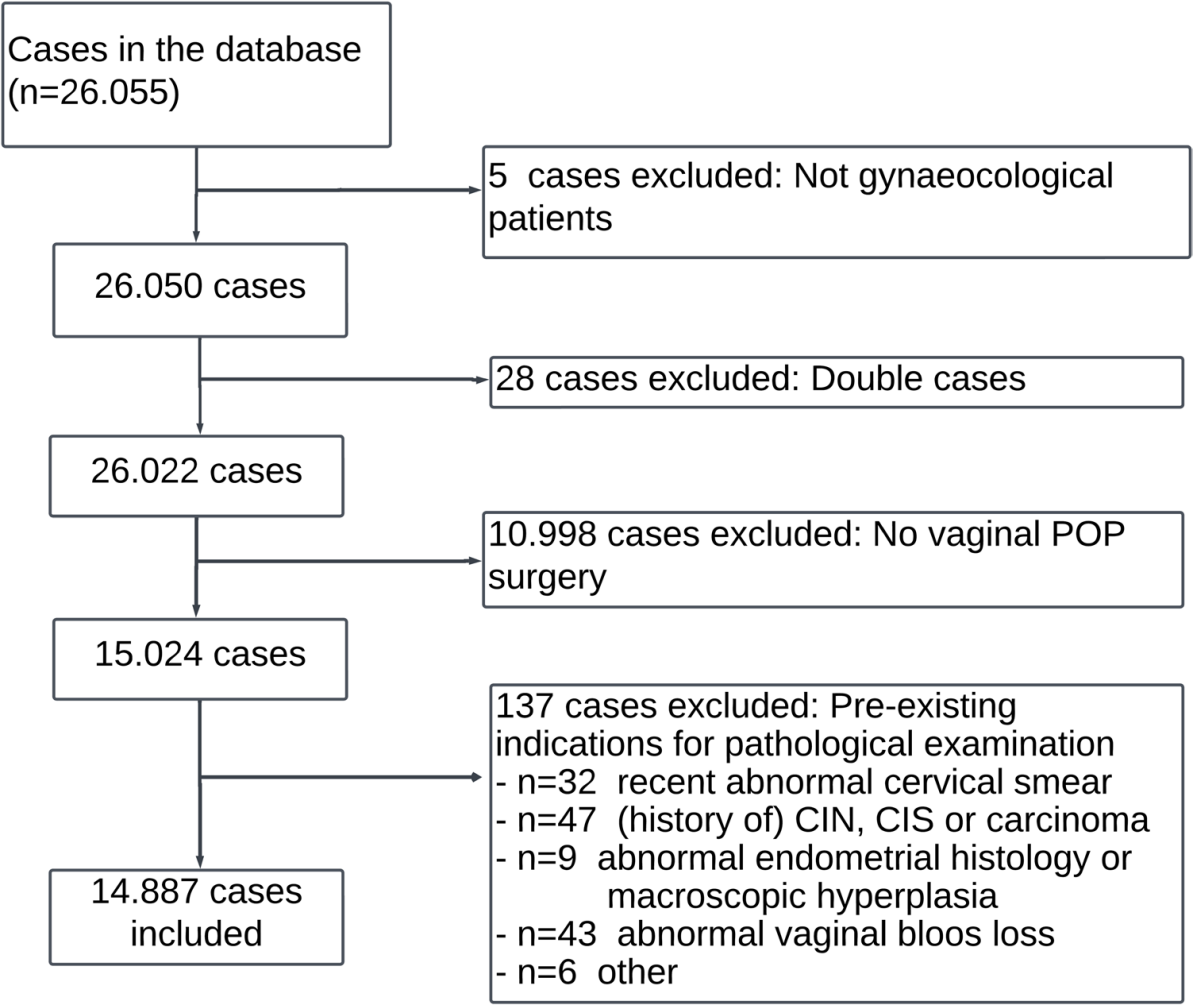
Patient inclusion diagram

In total, 14.887 patients were included in this study, with a median age of 61.4 years (SD = 11.7). See Fig. 1 for a visual representation.

In 11.978 (80.5%) cases, a previous cervical smear was available. In the other 19.5%, previous cervical smears were not included in our database. The mean time from the last cervical smear to prolapse surgery was 3.2 years (SD = 4.4).

### Histopathology

Among the *n* = 14.887 women, the overall risk of CIN or cervical malignancy was 2.0% [1.8, 2.3]. CIN I, II, and III were reported in *n* = 200 (1.3% [1.2, 1.5]), *n* = 56 (0.4% [0.3, 0.5]) and *n* = 46 (0.3% [0.2, 0.4]) of all cases, respectively. No cases of AIS were detected.

Furthermore, one cervical carcinoma was reported (0.007% [−0.0006, 0.02]); more specifically, it was characterized as a microinvasive squamous cell carcinoma. This cervical carcinoma can be classified as FIGO stage IA1. The distribution for cervical abnormalities was 66.0% CIN I, 18.5% CIN II, 15.2% CIN III, and 0.3% cervical carcinomas. No other malignancies were diagnosed.

A total of 103 incidental findings of CIN II+ were found, see Table 2. The overall prevalence of CIN II+ lesions was 6.9 [5.6, 8.3] per 1000 women, while cervical carcinomas were reported at 6.7 [−0.6, 19.9] per 100.000 women.

**Table 2:** Histopathological report: numbers and percentages of malignancies and pre-malignancies in women with cervical removal as part of pelvic organ prolapse

Histopathology	Cervical amputation (*n* = 14.892)	% [95%-CI]
**CIN II+**^**1**^	**103**	**0.69% [0.55, 0.82]**
CIN II	56	0.38% [0.28, 0.47]
CIN III	46	0.31% [0.22, 0.40]
**Malignancies**	**1**	**0.0067% [−0.0064, 0.020]**
*Cervical*	*1*	*0.0067% [−0.0065, 0.020]*
Squamous cell	1	0.0067% [−0.0064, 0.020]
Adenocarcinoma	0	0%
*Other*	*0*	*0%*
**Total**	**104**	**0.69% [0.56, 0.83]**

^1^Total number of CIN II and CIN III lesions, this includes detected AIS

In bold the total number of pre-malignancies and malignancies are shown. In italics the number of malignancies per origin are shown. Cervical origin is further devided in squamous cell and adenocarcinoma

The NNT for CIN II+ lesions and malignancies was 144.5 and 14.887 women, respectively.

## Discussion

This nationwide study observed a prevalence of CIN II+ lesions of 6.9 per 1000 women, among women undergoing cervical amputation as part of POP surgery. One solitary case of cervical malignancy was identified as an incidental finding.

### Results of the study in context of other observations

To our knowledge, there have been no studies published on incidental cervical abnormalities after amputation of the cervix as part of POP surgery. However, studies on cervical lesions found after hysterectomy as part of POP surgery have been performed, with prevalences ranging from 0.42 to 1.44% for CIN and cervical cancer, including CIN I [19–26]. Recommendations varied, ranging from maintaining routine examination to omitting it and relying solely on macroscopic assessment [19, 21, 24]. These prevalences are lower than the 2.03% in our study. This could be explained by the availability of more clinical data, and therefore excluding women more accurately in case of pre-existing indications for histological examination. Thus, excluding more women with a higher a priori probability of abnormal findings than in our study.

In 2021, 1.1% of all women participating in the population screening program for cervical cancer in the Netherlands were diagnosed with CIN II+ [12]. This incidence will presumably decrease due to the implementation of a national HPV vaccination program [27]. Our study showed several differences from the national screening program. The national detection rate was higher than the rate of 0.7% found in our study. Furthermore, the proportion of CIN II+ lesions in our population was lower than among the women in the nationwide screening program. In the screening program, the distribution of abnormal findings was 35.5% for CIN I, 28.9% for CIN II, 33.3% for CIN III, and 2.3% for cervical malignancies [12]. In our study, the distribution for cervical abnormalities was 66.0, 18.5, 15.2, and 0.3%, respectively. The differences in both prevalences and in the distribution of pathological findings may be attributed to variations in the target population: the screening program is for women aged 30–60 years, whereas our study included women with a mean age of 61.4 years (SD = 11.7). The national detection rate of CIN II+ lesions in women aged 60–64 years was reported to be <0.5% [28]. These findings substantiate questioning the need for routine examination: abnormalities in older populations are less likely to be CIN II+ lesions than in younger age groups [12, 29].

In this study, we focused on cervical amputation. Therefore, uterine malignancies may have been missed using uterine-sparing interventions. In Engelbredt et al. [14] 299 women were followed after uterine-conserving surgical treatment for a mean time of 7.8 years, in which only 6 patients developed abnormal histopathologic findings: *n* = 5 benign endometrial hyperplasia without atypia and *n* = 1 cervical intra-epithelial neoplasia (CIN I). This study indicates a low risk of developing uterine malignancy after uterine-sparing procedures.

Frick et al. additionally assessed the risk of unanticipated abnormal gynecological pathology after hysterectomy in *n* = 644 patients, both asymptomatic and symptomatic [30]. The symptomatic group underwent a preoperative evaluation using endometrial biopsy and/or ultrasound and were only included with a negative diagnostic evaluation. This study found unexpected endometrial cancer or hyperplasia in 3% of the asymptomatic postmenopausal women and in 13% of the symptomatic group. No (pre-)malignant uterine abnormalities were found in premenopausal women. Only two CINs were found, which both concerned a CIN I. Frick et al. [30] concluded that premenopausal women with either normal bleeding patterns or abnormal bleeding patterns with a negative preoperative evaluation were at minimal risk of incidental uterine disease. Their study recommended an endometrial biopsy and/or ultrasound screening preoperatively and advised against uterine preservation in postmenopausal women due to the 13% risk of unanticipated disease [30].

### Management of pathological findings

CIN I has high spontaneous regression rates (34–94%), and is therefore frequently observed rather than treated with excision or ablation [8, 10]. Due to these low progression rates, we reported clinically relevant (pre-)malignant findings as CIN II+ lesions.

For CIN II or III, Loop Excision of the Transformation Zone (LETZ) is recommended; therefore, a more comprehensive approach such as amputation of the cervix would also suffice [10]. The risk of progression to CIN III or carcinoma is between 3–11% for CIN I and 5–30% for CIN II [10]. CIN III has a 31.3% chance of progressing to invasive cervical cancer, but this risk can be reduced to 0.7% by adequate treatment with hysterectomy, amputation of the cervix, or cone/ring biopsy [31].

Diagnoses like micro-invasive carcinomas or other gynecological malignancies may require additional treatment depending on staging [10].

For early cervical cancer, up to FIGO stage IA2 without vasoinvasion, conisation is a sufficient treatment. However, more advanced stages require more extensive surgery, including pelvic lymph node dissection or even radiation therapy with or without chemotherapy [1, 9, 32].

In our study, all patients with CIN I-III (*n* = 302) received sufficient initial treatment through the performed cervical amputation [10]. The only malignancy found was a microinvasive cervical carcinoma with invasion of <1 mm, FIGO classification IA1, which was radically removed [1]. However, further follow-up would be indicated after removal of the (pre-)malignant findings [10, 33].

### Costs and environmental impact

Growing focus on rising healthcare costs and the healthcare contribution of up to 5% of the global environmental impact should also be taken into account in the consideration for routine histopathological examination [34, 35].

Carbon emission in histopathology can be attributed to, e.g., usage of consumables, instruments, and commuting personnel [36]. Given the limited opportunities for reducing the carbon footprint during the histopathological testing process, the greatest benefit can be accomplished by minimizing unnecessary testing [37].

Moreover, costs can be reduced by selectively performing routine histopathological examination. The expenses for cyto- and histopathological examination of the cervix in our hospital range from €55 to €150. While reducing the number of examinations after POP surgery may not completely alleviate the costs, since overhead costs are incurred for items such as space/rent, support staff, utilities, management, etc., the freed-up capacity can be directed to other areas.

### Strengths and limitations

The strength of this study lies in the large number of included patients and the extended timespan of inclusion, providing a more accurate representation of the prevalence of cervical pathology in time.

Furthermore, this study analyzed patients’ histories of cervical smears and cervical and endometrial histology to reliably exclude those with indication for histology before surgery.

However, there were limitations to this study. Firstly, data collection and analysis were based on the provided clinical information on the application form. If this information did not specify prolapse as an indication for surgery, the case was excluded from our study. Additionally, this study relied on the inclusion of symptoms, macroscopic abnormalities, and relevant patient history on the form. Based on the information supplied, patients with abnormal vaginal bleeding were excluded. However, if relevant clinical knowledge was not mentioned, symptomatic patients could potentially have been included in this study. Similarly, unreported patient history and the presence of macroscopic anomalies could have influenced the results of this study.

The applicability of this data to other populations could also be a limitation, due to the Dutch national screening program for cervical cancer. This screening program could reduce the risk of cervical malignancy by early detection of pre-cancerous lesions [12]. When assessing omitting routine histopathological examination, healthcare professionals should factor in the presence and quality of a national screening program for cervical cancer.

### Future

While the prevalence of cervical pathology found in this study was low, further research is necessary to assess patient safety regarding alternatives for histopathological examination, such as macroscopic assessment using acetic acid (VIA) or Lugol’s iodine (VILI) [24, 38]. Another alternative could be routine cervical smear testing, although the sensitivity of this method in screening for CIN and cervical cancer is around 74%, implying that 1 in 4 women with abnormalities could potentially be missed [39]. In addition, Chundarat et al. found a 4% false negative rate of cervical smears pre-hysterectomy for abnormal cervical histopathology [40].

Moreover, a comprehensive evaluation, including additional cost–benefit analyses and consideration of the environmental impact, is crucial to strike a balance between routine histopathological examination and the risk of missing unexpected (pre-)malignancies.

## Conclusion

This study found a prevalence of 0.7% for CIN II+ incidental findings in women undergoing amputation of the cervix as part of POP surgery. No additional treatment was required following the final histopathological results.

The decision to omit routine histopathological examination could potentially be safe, offering the prospect of reduced healthcare costs and environmental impact. Healthcare professionals should individually assess the risks and benefits of omitting and/or replacing routine histopathological examination.

## Data Availability

Data can be obtained from Palga: Dutch Pathology and Registry, under the name of 2020-3_update Prevalence and clinical relevance of cervix abnormalities after prolapse surgery.

## Abbreviations

POP: Pelvic organ prolapse
CIN: Cervical intraepithelial neoplasia
AIS: Adenocarcinoma in situ
LEEP: Loop electrosurgical excision procedures
CI: Confidence interval
NNT: Number needed to treat
SD: Standard deviation
